# Presence and rehabilitation: toward second-generation virtual reality applications in neuropsychology

**DOI:** 10.1186/1743-0003-1-9

**Published:** 2004-12-08

**Authors:** Giuseppe Riva, Fabrizia Mantovani, Andrea Gaggioli

**Affiliations:** 1 Applied Technology for Neuro-Psychology Lab., Istituto Auxologico Italiano, Milan, Italy; 2 Department of Psychology, Catholic University of Milan, Milan, Italy; 3 Department of Epistemology and Hermeneutics of Education, University of Milan-Bicocca, Milan, Italy; 4 Psychology Lab., Department of Preclinic Sciences LITA VIALBA, State University of Milan, Milan, Italy

## Abstract

Virtual Reality (VR) offers a blend of attractive attributes for rehabilitation. The most exploited is its ability to create a 3D simulation of reality that can be explored by patients under the supervision of a therapist. In fact, VR can be defined as an advanced communication interface based on interactive 3D visualization, able to collect and integrate different inputs and data sets in a single real-like experience.

However, "treatment is not just fixing what is broken; it is nurturing what is best" (Seligman & Csikszentmihalyi). For rehabilitators, this statement supports the growing interest in the influence of positive psychological state on objective health care outcomes.

This paper introduces a bio-cultural theory of presence linking the state of optimal experience defined as "flow" to a virtual reality experience. This suggests the possibility of using VR for a new breed of rehabilitative applications focused on a strategy defined as *transformation of flow*. In this view, VR can be used to trigger a broad empowerment process within the flow experience induced by a high sense of presence.

The link between its experiential and simulative capabilities may transform VR into the ultimate rehabilitative device. Nevertheless, further research is required to explore more in depth the link between cognitive processes, motor activities, presence and flow.

## Introduction

What is virtual reality (VR)? For many health care professionals, VR is first of all a technology. Since 1986, when Jaron Lamier used the term for the first time, VR has been usually described as a collection of technological devices: a computer capable of interactive 3D visualization, a head-mounted display and data gloves equipped with one or more position trackers.

But is this definition enough to describe the potential of VR for rehabilitation? If we look at its actual applications in rehabilitation, the answer is probably not [1].

VR can be considered the leading edge of a general evolution of present communication interfaces like television, computer and telephone [2]. The main characteristic of this evolution is the full immersion of the human sensorimotor channels into a vivid and global communication experience [3].

In fact, VR is used in rehabilitation as "an advanced form of human-computer interface that allows the user to interact with and become immersed in a computer-generated environment in a naturalistic fashion" [4].

Following this vision Rizzo *et al. *[5] identify twelve assets that are available with VR for neuropsychological applications:

• The capacity to systematically deliver and control dynamic, interactive 3D stimuli within an immersive environment that would be difficult to present using other means.

• The capacity to create more *ecologically *valid assessment and rehabilitation scenarios.

• The delivery of immediate performance feedbacks in a variety of forms and sensory modalities.

• The provision of "cueing" stimuli or visualization tactics designed to help guide successful performance to support an error-free learning approach.

• The capacity for complete performance capture and the availability of a more naturalistic/intuitive performance record for review and analysis.

• The capacity to pause assessment, treatment and training for discussion and/or integration of other methods.

• The design of safe testing and training environments that minimize the risks due to errors.

• The capacity to improve availability of assessment and rehabilitation by persons with sensorimotor impairments via the use of adapted interface devices and tailored sensory modality presentations built into VE scenario design.

• The introduction of "gaming" features into VR rehabilitation scenarios as a means to enhance motivation.

• The integration of virtual human representations (avatars) for systematic applications addressing social interaction.

• The potential availability of low cost libraries of VEs that could be easily accessed by professionals.

• The option for self-guided independent testing and training by clients when deemed appropriate.

In summary, VR provides a new human-computer interaction paradigm in which users are no longer simply external observers of images on a computer screen but are active participants within a computer-generated three-dimensional virtual world [6-8]: in the virtual environment (VE) the patient has the possibility of learning to manage a problematic situation related to his/her disturbance in a functionally relevant, ecologically valid experience [9,10].

This outline better clarifies the possible role of VR in rehabilitation: a communication interface based on interactive 3D visualization, able to collect and integrate different inputs and data sets in a single real-like experience [2,11].

This is possible because the key characteristic of VR, differentiating it from other media or communication systems, is the sense of *presence *[12], usually defined as the "sense of being there" [13], or the "feeling of being in a world that exists outside the self" [14].

This feeling is theorized to contribute to the efficacy of VR as rehabilitation tool: the successful use of VR exposure therapy for phobias [15-18], postraumatic stress disorders [19-21], and the pain reduction obtained in burn patients during a VR session [22-25] underline the possible role that an high level of presence, elicited by the VR experience, may have in the rehabilitation process.

Thanks to presence, not only knowledge acquisition is possible in VR, but also this acquired knowledge can be transferred in a real environment [26,27]. This, evidence, coming also from different neuropsychological studies [1], adds value on VR use in the rehabilitation of highly social disabling cognitive functions, highlighting how goals reached in controlled settings may be transferred on patients' everyday life.

Now, the challenge within this area is the creation of new paradigms [28]. As clearly underlined by Morganti [1] "More than a playing tool supporting cognitive or motor performances VR simulation has to provide a powerful chance to build personal meaning, map and strategies interacting with it."

Within this context we propose to investigate the impact of VR on rehabilitation and subjective experience from a theoretical perspective that stresses the active role of individuals in interacting with their natural and cultural environment [29,30]. In this process a key role is played by the concept of "presence" and its link with our optimal experiences.

## A bio-cultural theory of presence

### Presence as separation between "external" and "internal"

What is presence? Answering to this question is not a simple task [12,31,32]. In fact, if we check the present status of presence research, we can find two different but coexisting visions.

A first group of researchers describes the sense of presence as a function of our experience of a given medium [33-41]. The main result of this approach is the definition of presence as the *perceptual illusion of non*-*mediation *[37], produced by means of the disappearance of the medium or its transformation into a social entity. Following this vision the experience of presence is only related to our interaction with an external artifact. The main advantage of this approach is its predictive value: the level of presence is reduced by the experience of mediation during the interaction.

This approach, however, does not address some broader questions: What is presence for? Is it a specific cognitive process? What is its role in our daily experience?

To answer these questions a second group of researchers considers presence as a neuropsychological phenomenon, evolved from the interplay of our biological and cultural inheritance, whose goal is the control of agency [2,12,42-49].

Within this paper, we will support the second vision, trying to link it with the outcome of the first one [50]. Here, presence is delineated as an evolved bio-cultural internal selection mechanism that helps the self in organizing the streams of sensory data: the more it can differentiate the self from the external world, the more is our experience of presence. The main goal of this differentiation is the control of agency to improve the possibility of survival within the external environment.

To fully understand the key ideas behind this vision, three points are critical:

– Presence has a simple but relevant role in our everyday experience: the control of agency through the unconscious separation of "internal" and "external". The meaning of "internal" and "external" is not related only to the body but also to the social and cultural space (situation) in which the self is in;

– The presence-as-process (the separation mechanism) produces, but it is different from the presence-as-feeling (the experienced level of presence);

– The presence-as-feeling is experienced indirectly by the self through the characteristics of action and experience. In fact the self perceives directly only the variations in level of presence-as-feeling: breakdowns and optimal experiences.

First, presence is described here as a defining feature of self and it is related to the evolution of a key feature of any central nervous system: the embedding of sensory-referred properties into an internal functional space. As noted by Waterworth and Waterworth [47], the appearance of the sense of presence allows the nervous system to solve a key problem for its survival: how to differentiate between internal and external states. Without the emergence of the sense of presence it is impossible for the nervous system to experience *distal attribution *– the referencing of our perception to an external space beyond our boundaries – and effectively control its agency.

In this vision it is important to distinguish between presence-as-process and presence-as-feeling. The presence-as-process is the continuous activity of the brain, organized around the three functionally and phylogenetically different layers discussed in the next paragraph, in separating "internal" and "external" within different kinds of afferent and efferent signals.

A critical point here is to explain why we need to introduce a new cognitive process – presence-as-process – to monitor our activity. The answer comes from a recent paper by de Vignemont and Fourneret [51]. These authors discuss the position of Wittgenstein [52] about agency. According to this author, agency involves a primitive notion of the self as subject, which does not rely on any prior perceptual identification and which is immune to error through misidentification.

However, both the neuroscience of action and the neuropsychology of schizophrenia are countering his position [53]. For instance, the analysis of positive deficits underlying positive symptoms in schizophrenia has shown that is not possible to reduce the sense of agency to action control or action awareness. To overcome this problem, de Vignemont and Fourneret distinguish in agency between the sense of initiation and the sense of one's own movements. As they underline [51] "the double sense of agency depends on the same mechanisms of action control: it results from the unconscious comparison between different kinds of afferent and efferent signals. Therefore, these monitoring systems allow one to automatically distinguish one's own actions and those of the other" (p. 15). So, presence-as-process can be described as a sophisticated form of monitoring of action and experience, transparent to the self but critical for its existence.

As clarified by Russell [54]: "Action-monitoring is a subpersonal process that enables the subjects to discriminate between self-determined and world-determined changes in input. It can give rise to a mode of experience (the experience of being the cause of altered inputs and the experience of being in control) but it is not itself a mode of experience." (p.263).

For this reason, the presence-as-feeling (level of presence) is not separated from the experience of the subject but it is related to the quality of our actions. It corresponds to what Heidegger [55] defined "the interrupted moment of our habitual standard, comfortable *being*-*in*-*the*-*world*" In fact, a higher level of presence-as-feeling is experienced by the self as a better quality of action and experience [46,56]. However, the self becomes aware of the presence-as-feeling separated by our *being*-*in*-*the*-*world *when its level is modified. More in detail, the self perceives directly only *the variations *in the level of presence-as-feeling: breakdowns and optimal experiences.

The process of adaptation to the natural environment provided humans with specific biological features, such as the upright position, the opposing thumb and the increase in brain mass that allowed survival and reproduction in any environmental niche. At the same time, by means of the differential investment of attention and psychic resources, the individual selects and organizes the information acquired from his/her context according to an emergent, autonomous criterion: the quality of experience [57,58]. In our view, another evolutionary goal of presence-as-process is to track the quality of experience identifying highs and lows.

On one side we have optimal experiences. According to Csikszentmihalyi [59,60], individuals preferentially engage in opportunities for action associated with a positive, complex and rewarding state of consciousness, defined optimal experience or flow. Here we suggest that flow is the result of the link between the highest level of presence-as-feeling, with a positive emotional state. In fact, it is also possible to experience the highest level of presence together with negative emotional states: e.g. in the battlefield during an attack from the enemy.

On the other side we have breakdowns. Winograd and Flores [61] refer to presence disruptions as *breakdowns: a breakdown *occurres when, during our activity, an object or an environment becomes part of our consciousness. If this happens, we shift our attention from action to the object/environment to cope with it: e.g., when a wall stops our movement.

Why do we experience these breakdowns? Our hypothesis is that breakdowns are a sophisticated evolutionary tool used by the presence-as-process to control the quality of experience: the more the breakdown, the less is the level of presence-as-feeling, the less is the quality of experience, and the less is the possibility of surviving in the environment.

The importance of breakdowns for understanding presence is well reflected by Slater's concept of "break in presence" (BIP) [62]: a break in presence is the moment of switch between responding to signals with source in environment X to those with source in environment Y. In a BIP the critical issue is how will the actor act? To which set of signals will the actor respond?

The answers to these questions are related to another important point of our vision: the meaning of "internal" and "external". In our vision, the boundaries are not only physical and related to our body (being there), but also social and cultural (making sense there). As underlined by Slater [63], Presence "is the *total response *(italics in the original) to being in a place, and to being in a place with other people. The 'sense of being there' is just one of many signs of presence – and to use it as a definition or a starting point is a category error: somewhat like defining humor in terms of a smile" (p. 7).

If, in relatively simple organisms, this separation involves only a correct coupling between perceptions and movements, in humans it also implies the relation of the subject with a social and cultural space [44,64]. In fact, individuals actively interact with the environment, selecting and differentially replicating throughout their lives a subset of biological and cultural information, in terms of activities, interests and values. This vision has two important corollaries:

– it is also "external" to the subject what is not related to his/her activities, interests and values.

– to be more "present" in the situation (social and cultural space) defined by a symbolic system, the user has to be aware of its meaning. Only "making sense there", the user really experiences a full sense of presence. In giving sense to a situation an important role is usually played by narratives [56,65].

To make these concepts clearer an example may help. I'm in a restaurant for a formal dinner with my boss and some colleagues, but I don't know how to use the many different strange forks I have around my dish. In this situation I'm physically there, but the lack of knowledge puts me outside, at least partially, from the social and cultural space of the "formal dinner". The result is a limitation in my agency: I don't use the forks to avoid mistakes. This example shows clearly how both physical boundaries (wall, obstacles, etc.) and social and cultural boundaries have a strong influence on the possibility of action and the quality of experience of the subject.

At this point our conclusions are:

• *In the real world*, *the feeling of presence is not the same in all the situations but can be different in relation to the characteristics of the social and cultural space the subject is in*. For instance, if I'm attending a lesson in university, my level of presence can be lower or higher in relation to the interest I have in the topic discussed. If the lesson is totally boring I can be "absent" (totally internal): in absence attention is mostly directed towards internally-generated scenarios (in imagination) which are not currently present in the world [43]. The role of "absence" is critical for the survival of the subject. Is in fact in absence that the subject defines plans and organizes future behaviors.

• There are some exceptional situations in real life in which the activity of the subject is characterized by a higher level of presence. In these situations the subject experiences a full sense of control and immersion. When this experience is associated to a positive emotional state, it can create an optimal experience, usually defined "flow". An example of flow is the case where a professional athlete is playing exceptionally well (positive emotion) and achieves a state of mind where nothing else matters but the game (high level of presence).

### The layers of presence

At this point we have defined what presence is and its role in the human experience. However, there is yet another open question: how can we achieve high level of presence-as-feeling? To answer this question we have to analyze the neuropsychological nature of presence-as-process.

Even if presence is a unitary feeling, on the process side it can be divided in three layers/subprocesses (for a broader and more in-depth description see [50]). These layers are phylogenetically different, and strictly related to the three levels of self identified by Damasio [66]:

• *The proto self*: a coherent collection of neural patterns that map, moment by moment, the physical state of the organism;

• The *core self*: a transient entity which is continuously generated through encounters with objects

• The *extended self*: a systematic record of the more invariant properties that the organism has discovered about itself.

Each layer of presence solves a particular facet of the internal/external world separation and it is characterized by specific properties. In particular we can make conceptual distinctions between *proto presence (self vs. non self)*, *core presence (self vs. present external world)*, and *extended presence (self relative to present external world)*.

More precisely we can define **proto presence **as an *embodied presence related to the level of perception-action coupling (self vs. non-self)*. The more the organism is able to couple correctly perceptions and movements, the more it differentiates itself from the external world, thus increasing its probability of surviving. Proto presence is based on proprioception and other ways of knowing bodily orientation in the world. In a virtual world this is sometimes known as "spatial presence" and requires the tracking of body parts and appropriate updating of displays.

**Core presence **can be described as *the activity of selective attention made by the self on perceptions (self vs. present external world)*: the more the organism is able to focus on its sensorial experience by leaving in the background the remaining neural processes, the more it is able to identify the present moment and its current tasks, increasing its probability of surviving. Core presence is based largely on vividness of perceptible displays. This is equivalent to "sensory presence" (e.g. in non-immersive VR) and requires good quality, preferably stereographic, graphics and other displays features.

Finally, the role of **extended presence **is to *verify the significance to the self of the events experienced in the external world (self relative to the present external world)*. The more the self is present in significant experiences, the more it will be able to reach its goals, increasing the possibility of surviving. Extended presence requires intellectually and/or emotionally significant content.

In humans the sense of presence-as-feeling is a direct function of these three layers: the more they are able to separate between "internal" and "external", the more is the feeling of presence, the better is the quality of action and experience.

## VR, presence and flow

A corollary of the proposed vision is critical for our goals: it is possible to design mediated situations that elicit exceptionally high presence [67-69].

In particular, here we argue that virtual reality is the medium able to support the highest level of presence because it can trigger at the same time all the three layers discussed before. To understand this point and in particular the difference between VR and other media, here are some examples [50]:

• In reading an engrossing book while sitting in a comfortable, safe place, extended presence will be engaged by media (engagement) but the other layers will not be involved.

• In looking at a movie, you can activate a high level of core presence (vividness), a high level of extended presence (engagement) but no proto presence (spatial presence).

• In experiencing an interesting immersive VR experience, proto (spatial presence), core presence (vividness) and extended presence (engagement) will be activated by the medium.

• In an immersive VR experience, if you are pre-occupied with personal worries and the mediated content is not very engaging, proto (spatial) and core presence (vividness) will be invoked by the medium, but not extended presence.

The possibility of activating all the three layers at the same time reduces the occurrence of breakdowns. As suggested by Marsh and colleagues [41], one of the main goals of VR is to maintain users' attention in the content/illusion of a VR system. The final result is the *perceptual illusion of non*-*mediation *[37], produced by means of the disappearance of the medium, which activates the highest level of presence.

To achieve an optimal experience, however, a next step is required: the highest level of presence has to be linked to a positive emotional experience.

Csikszentmihalyi [60,70] defines "flow" as an optimal state of consciousness characterized by a state of concentration so focused that it amounts to absolute absorption in an activity. According to Csikszentmihalyi [71] when people are in a flow state " [they] shift into a common mode of experience when they become absorbed in their activity. This mode is characterized by a narrowing of the focus of awareness, so that irrelevant perceptions and thoughts are filtered out; by loss of self-consciousness; by a responsiveness to clear goals and unambiguous feedback; and by a sense of control over the environment it is this common flow experience that people adduce as the main reason for performing the activity" (p72).

Starting from this definition, different authors tried to define flow in an operational way. For Ghani and Deshpande [72] the two main characteristics of flow are (a) the total concentration in an activity and (b) the enjoyment which one derives from the activity. Moreover, these authors identified two other factors affecting the experience of flow: a sense of control over one's environment and the level of challenge relative to a certain skill level.

In this paper we suggest that VR is the preferred medium for the activation of the flow experience. A number of recent experimental results might be considered to foster this vision. A first support comes from the work of Hoffman and his group in the treatment of chronic pain [22-25]. Few experiences are more intense than the pain associated with severe burn injuries. In particular the daily wound care – the cleaning and removal of dead tissue to prevent infection – can be so painful that even the aggressive use of opioids (morphine-related analgesics) cannot control the pain. However it is well known that distraction – for example, by having the patient listen to music – can help to reduce pain for some people. So, Hoffman and colleagues verified in a controlled study the efficacy of VR as an advanced distraction by comparing it with a popular Nintendo video game. The result showed dramatic drops in pain ratings during VR compared to the video game [73]. Further, using functional magnetic resonance imaging (fMRI) scanner they measured pain-related brain activity for each participant during conditions of no virtual reality and during virtual reality (order randomized). The team studied five regions of the brain that are known to be associated with pain processing – the anterior cingulate cortex, primary and secondary somatosensory cortex, insula, and thalamus – and found that during VR the activity in all the regions showed significant reductions. In particular, the results showed direct modulation of human brain pain responses by VR distraction: the amount of reductions in pain-related brain activity ranged from 50 percent to 97 percent.

A second set of results comes from the work of Gaggioli [29,30]. Gaggioli compared the experience reported by a user immersed in a virtual environment with the experience reported by the same individual during other daily situations. To assess the quality of experience the author used a procedure called Experience Sampling Method (ESM), which is based on repeated on-line assessments of the external situation and personal states of consciousness [74]. Results showed that VR experience was the activity associated with the highest level of optimal experience (22% of self-reports). Reading, TV viewing and the use of other media – both in the context of learning or leisure activities – obtained lower percentages (respectively 15%, 8% and 19% of self-reports) of optimal experiences.

A final result, is the preference of phobic patients for VR vs. traditional treatments as showed by two studies from García-Palacios and colleagues [75,76]. In their last study, which surveyed 102 patients who met DSM-IV criteria for specific phobias or panic disorder with agoraphobia, 70% of the patients asked to choose between "in vivo" exposure vs. VR exposure therapy, chose VR exposure. Further, 23.5% of the sample refused in vivo exposure whereas only 3% refused VR treatment.

## Presence and optimal experience: towards second-generation VR applications in rehabilitation

Authentic rehabilitation implies the active participation of the patient in the cultural context, their exposure to opportunities for action and development, their freedom to select opportunities they perceive as the most challenging and meaningful ones for the subject [29,77]. Following this vision, another important asset potentially offered by VR to the rehabilitation process is the possibility of triggering optimal experiences [78].

Optimal experiences promote individual development. As underlined by Massimini and Delle Fave, [58] "To replicate it, a person will search for increasingly complex challenges in the associated activities and will improve his or her skill, accordingly. This process has been defined as *cultivation*; it fosters the growth of complexity not only in the performance of flow activities but in individual behavior as a whole." (p. 28).

This process can be activated also after a major trauma. As noted by Delle Fave [79], to cope with dramatic changes in the daily life and in the access to environmental opportunities for action, individuals may develop a strategy defined as *transformation of flow*. Where possible, they keep cultivating former flow activities. Otherwise, as often happens, they manage to identify new and unexpected sources of concentration and involvement, sometimes in areas very different from their previous interests.

The vision behind the concept of transformation of flow is the one from "Positive Psychology" [80]. According to this vision, existing professional treatments should include therapeutic factors that are related to positive experiences. These include increasing clients' positive expectations and hope about change, general sense of optimism, self-efficacy, and coping strategies. Numerous studies of patients with life-threatening diseases suggest that those who remain optimistic show symptoms later and survive longer than patients who confront reality more objectively [81]. That is, rehabilitative treatments should also be evaluated in terms of their ability to make life more fulfilling for clients. However, it is very difficult within the traditional rehabilitative practices to cope with the sense of hopefulness and depression expressed by many patients.

In this area VR may offer a critical advantage: the possibility for the patient to manage successfully in a VE a problematic situation related to his/her disturbance. Using VR in this way, the patient is more likely not only to gain an awareness of his/her need to do something to create change but also to experience a greater sense of personal efficacy [82]. This approach was recently tested in the support of cerebral palsy. More in detail, Miller and Reid [83] investigated the personal experiences of 19 children aged 8–11 with cerebral palsy involved in a virtual reality play intervention program. The results showed that children perceived engagement and flow in the virtual reality, and increased their self-competence and self-efficacy. Further, they experienced a sense of control and mastery over the virtual environment and perceived physical changes and increased social acceptance from both peers and family.

In another case study, Riva [84] tested the possibility of using a VE experience – a stroll through a mountain path to reproduce the feeling of an excursion to the mountains – to support the rehabilitation of a person with spinal cord injury. The results revealed slightly improved levels of self-confidence, will, relaxation, and activity. The patient also declared subjective improvement in his sense of well-being, mood, and quality of sleep.

Generally, these techniques can be used as triggers for a broader empowerment process within the flow experience induced by a high sense of presence. In psychological literature *empowerment *is considered a multi-faceted construct reflecting the different dimensions of being psychologically enabled, and is conceived of as a positive additive function of the following three dimensions [85]:

– *perceived control*: includes beliefs about authority, decision-making skills, availability of resources, autonomy in the scheduling and performance of work, etc;

– *perceived competence*: reflects role-mastery, which besides requiring the skillful accomplishment of one or more assigned tasks, also requires successful coping with non-routine role-related situations;

– *goal internalization*: this dimension captures the energizing property of a worthy cause or exciting vision provided by the organizational leadership.

Virtual reality may be considered the preferred environment for the empowerment process, since it is a special, sheltered setting where patients can start to explore and act without feeling threatened. In this sense the virtual experience can be described as an "empowering environment" that rehabilitation provides to patients: nothing the patient fears can "really" happen to them in VR. With such assurance, they can freely explore, experiment, feel, live, and experience feelings and/or thoughts. VR thus becomes a most useful intermediate step between the therapist's office and the real world. Within this frame, therapists are encouraged to explore whether and how VR induced optimal experiences may facilitate recovery [86].

The use of VR as an empowerment tool was recently tested in the support of HIV-AIDS patients [87]. The system implemented a VR walkthrough experience of a relaxing campfire in a forest. The scene contains four interactive avatars that relate narratives compiled from HIV/AIDS patients. These narratives cover the aspects of receiving an HIV+ diagnosis, intervention, and coping with living with HIV+ status.

In terms of emotional impact, the participants found their experience with the system mostly encouraging, particularly the narratives relating to adjustment and coping.

## Challenges and Open Issues

VR is an advanced communicative interface based on interactive 3D visualization. Its simulative capabilities allow for the precise presentation and control of dynamic multi-sensory 3D stimulus environments, as well as providing advanced methods for recording behavioral responses.

However VR, differently from other media, can induce a high sense of *presence *[12], usually defined as the "sense of being there" [13], or the "feeling of being in a world that exists outside the self" [14]. Thanks to presence, not only knowledge acquisition is possible in VR, but also this acquired knowledge can be transferred in a real environment.

This paper introduces a bio-cultural theory of presence linking the state of optimal experience, defined as "flow", to a virtual reality experience.

The key ideas behind the proposed vision of presence are:

– Presence has a simple but critical role in our everyday experience: the control of agency through the unconscious separation of "internal" and "external". The meaning of "internal" and "external" is not related only to the body but also to the social and cultural space (situation) in which the self is in.

– The presence-as-process (the separation mechanism) produces, but is different from, the presence-as-feeling (the experienced level of presence);

– The presence-as-feeling is experienced indirectly by the self through the characteristics of action and experience. In fact the self perceives directly only the variations in level of presence-as-feeling: breakdowns and optimal experiences;

– The presence-as-process can be divided in three different layers/subprocesses. They are phylogenetically different, and strictly related to the three levels of self identified by Damasio: *proto presence (self vs. non self)*, *core presence (self vs. present external world)*, and *extended presence (self relative to present external world)*.

A corollary of the proposed vision is the possibility to design mediated situations that elicit exceptionally high presence. In particular, here we argued that virtual reality is the medium able to support the highest level of presence because it can activate at the same time all the three layers. To achieve an optimal experience (flow), however, a next step is required: the highest level of presence has to be linked to a positive emotional experience.

The link between presence, flow and VR suggests the possibility of using it for a new breed of rehabilitative applications focused on a strategy defined as *transformation of flow*. In this view, VR can be used to trigger a broad empowerment process within the flow experience induced by a high sense of presence. Linking this possibility to its simulative capabilities may transform VR in the ultimate rehabilitative device.

Applications of VR in rehabilitation include the following disturbances: memory disorders, planning and motor disabilities, executive functions and spatial knowledge disabilities. For a full review, see Morganti [1]. However, the road is still long.

Even if the significant advances in computer and graphic technology drastically improved the characteristics of a typical VE, VR is still limited by the maturity of the systems available. Today, no off-the-shelf solutions are available. So, the set up of a VR system usually requires much patience for dealing with conflicting hardware or lacking drivers. Nearly every VR system requires a dedicated staff or at least a computer technician to keep the system running smoothly.

Moreover, introduction of patients and clinicians to VEs raises particular safety and ethical issues. In fact, despite developments in VR technology, some users still experience health and safety problems associated with the use of immersive headsets. Generally, for a large proportion of VR users these effects are mild and subside quickly.

Further, even if the clinical rationale behind the use of VR in rehabilitation is now clear, much of this research growth has been as feasibility studies and pilot trials. Hence, there is still limited convincing evidence coming from controlled studies.

Why there are so few controlled trials in VR research? The possible answers are two.

First, there is a lack of standardization in VR devices and software. To date, very few of the various VR systems available are interoperable. This makes difficult their use in contexts other than those in which they were developed. Second, there is a lack of standardized protocols that can be shared by the community of researchers.

Clearly, building new and additional virtual environments is important so therapists will continue to investigate applying these tools in their day-to-day clinical practice. In fact, in most circumstances, the clinical skills of the rehabilitator remain the key factor in the successful use of VR systems.

Future research should explore how to develop VR environments able to provide the degree of challenge required to elicit the optimal experience. Further, research should also deepen the analysis of the link between cognitive processes, motor activities, presence and flow. This will allow a new generation of VEs in which the added value of VR is not only simulation and control.
